# Changing characteristics of medical directors in mental health trusts in England

**DOI:** 10.1192/bjb.2025.10123

**Published:** 2026-06

**Authors:** Peter L. Cornwall, Penny Walton

**Affiliations:** 1 Redcar & Cleveland Mental Health Services, Tees, Esk & Wear Valleys NHS Foundation Trust, Redcar, UK; 2 Newcastle University Medical School, Newcastle upon Tyne, UK

**Keywords:** Medical leadership, mental health services, senior medical leader, women, international medical graduates

## Abstract

**Aims and method:**

The medical workforce in psychiatry is increasingly diverse, but not necessarily in its senior leadership in the UK’s National Health Service (NHS). We aimed to describe the characteristics of psychiatrists with board-level responsibility in mental health trusts in England in 2024, comparing the current picture with that of 2016 and 2020, using publicly available data.

**Results:**

The proportion of medical directors who are female has not changed, so women remain underrepresented, while the proportion who are international medical graduates has increased substantially, so they are no longer underrepresented. Although fewer in number, intellectual disability psychiatrists are underrepresented.

**Clinical implications:**

Greater attention will need to be paid to developing female medical leaders if representative leadership is to be achieved.

Diversity in the National Health Service’s (NHS) leadership is a goal that has been repeatedly highlighted over recent years. Evidence has shown that having a diverse workforce is essential when addressing health inequalities within the patient group that they provide care for.^1^ It is clear that compassionate leadership with a focus on supporting and respecting each other leads to increased staff engagement and motivation, which in turn improves quality of patient care: ‘for leadership to be compassionate, it must also be inclusive’.^2^ As well as improving patient health outcomes, there is evidence that a diverse leadership team correlates with better NHS financial performance.^3^

Women attained parity in medical school admissions in the UK and elsewhere several decades ago, but we know they have been underrepresented in senior leadership positions.^4^ The NHS workforce race equality standard was introduced in 2015 to ensure employees from Black and minority ethnic backgrounds got equal access to career development.^5^ Since then the NHS has highlighted the need to improve the diversity of senior leadership teams and noted the need to give international recruits parity of access to career development.^6–8^

Data from research we conducted in 2016 and 2020 had previously shown that medical directors on NHS mental health trust boards who had studied in UK and Irish universities were overrepresented compared with doctors from international medical backgrounds.^9,10^ In the current study we aimed to describe the characteristics of psychiatrists with board-level responsibility in mental health trusts in England in 2024 using publicly available data, comparing the current picture with that of 2016 and 2020 and noting any changes.

## Method

We collected data on psychiatrists with board-level responsibility in 51 NHS trusts providing mental health services in England using a range of publicly available sources: trust websites and annual reports, the General Medical Council (GMC) medical register and the Royal College of Psychiatrists’ public members list. We included doctors with a specialist registration in psychiatry who were described as board directors, whether or not they had voting rights. Although they are not the only providers of NHS mental healthcare in England, at the time of data collection, almost the whole country was covered by 1 of 51 mental health trusts. There was one exception, namely the Isle of Wight NHS Trust, which was the sole provider of secondary care services on the island. Mental health services there are now provided by Hampshire and Isle of Wight Healthcare NHS Foundation Trust following the merger of Southern Health and Solent NHS Trusts, which reduced the number of mental health trusts from 51 to 50 in October 2024. Consequently, the number of trusts we examined was 51, and for sake of simplicity we refer to them all as mental health trusts. We restricted the analysis to England to replicate our earlier work and because England’s NHS trusts have a common structure, with the statutory requirement to have a medical director as a voting board member. At any point, a number of executive directors will hold the role in an interim or acting capacity, and we included them in the analysis.

The information collected comprised sex, date and country of primary medical qualification (PMQ), specialist registration, and date of board appointment. We categorised region of PMQ as the UK, European Economic Area (EEA) or international medical graduate (IMG). Specialist registration in the UK is in one of six specialties: general adult psychiatry, child and adolescent psychiatry, old age psychiatry, psychiatry of intellectual disability (also called learning disability psychiatry in the NHS), forensic psychiatry and medical psychotherapy.

We compared the current position with the results from our studies in 2016 and 2020.^9,10^ We divided the current sample into those appointed before and after 2021 in order to determine whether recently appointed directors had different characteristics from those appointed earlier. If a doctor had moved to a new position on the board, or in a different trust, we included them in the earlier appointed group.

To determine whether the doctors were representative of their peers in the consultant psychiatric workforce, we used the GMC register of doctors with a licence to practise in England and a specialist registration in psychiatry as a proxy for the NHS consultant psychiatrist workforce.^11^ We only included doctors who qualified in the PMQ year range of the board director group (1982–2008) to calculate the expected proportions.

Data analysis was undertaken using Real Statistics for Excel (version 365 for Windows).^12^ Categorical variables were compared using the *χ* ^2^-test. We used the *z*-statistic to evaluate whether the observed proportions were different from the actual proportions in the specialist workforce and used the normal distribution approximation to obtain confidence intervals (CIs) for the observed proportions.^12,13^

## Results

In July 2024, there were 55 psychiatrists and 6 non-psychiatrist doctors working as executive directors in the 51 mental health trusts in England (Table 1). Five of the non-psychiatrists were medical directors and comprised two paediatricians, one anaesthetist, one radiologist and one geriatrician. The remaining director was a general practitioner (GP) holding the post of chief operating officer. The median time from PMQ to board-level appointment was 25 years (range 12–38 years). The comparative figures for 2020 and 2016 were 25 years (range 14–43 years) and 24 years (range 13–33 years) respectively. Of the 55 psychiatrists, 43 were medical directors, 6 were joint medical directors and 3 were chief executive officers (CEO). The remaining three comprised a chief operating officer, a chief quality officer and a chief transformation officer. The number of psychiatrists working as chief executives has remained low, and in the past 8 years, 11 different trusts have employed a psychiatrist as CEO. All of the current CEOs were previously medical directors, with two out of the three promoted within their own trust. There has also been a major change in terminology, with the role of medical director now being described as chief medical officer in 31 trusts, compared with only 5 in 2020.

**Table 1:** Changes in the profile of board-level psychiatrists in England’s mental health trusts, 2016–2024

	2016	2020	2024	*χ* ^2^	d.f.	*P*
Mental health trusts, *n*	57	55	51			
Medical directors, *n*	60	57	54			
Psychiatrist medical directors, *n* (%)	55 (91.7)	49 (86.0)	49 (90.7)	1.14	2	0.56
Non-psychiatrist medical directors, *n* (%)	5 (8.7)	8 (14.0)	5 (9.3)			
Psychiatrists, *n*	63	54	55			
Medical directors, *n* (%)	49 (77.8)	46 (85.2)	43 (78.2)	2.15	6	0.91
Joint medical directors, *n* (%)	6 (9.5)	3 (5.5)	6 (10.9)			
Chief executive officers, *n* (%)	6 (9.5)	3 (5.5)	3 (5.5)			
Other, *n* (%)	2 (3.2)	2 (3.7)	3 (5.5)			
Sex						
Male, *n* (%)	44 (69.8)	37 (68.5)	40 (72.7)	0.24	2	0.88
Female, *n* (%)	19 (30.2)	17 (31.5)	15 (27.3)			
PMQ region						
UK, *n* (%)	42 (66.7)	34 (63.0)	27 (49.1)	56.0	4	<0.0001
IMG, *n* (%)	14 (22.2)	16 (29.6)	24 (43.6)			
EEA, *n* (%)	7 (11.1)	4 (7.4)	4 (7.3)			
PMQ IMG region						
South Asia, *n* (%)	7 (50.0)	11 (68.8)	16 (66.7)	17.21	4	<0.01
Africa, *n* (%)	6 (42.9)	4 (25.0)	8 (33.3)			
Other	1 (7.1)	1 (6.3)	0 (0.0)			
Specialist registration						
General adult psychiatry, *n* (%)	29 (46.0)	20 (37.0)	33 (60.0)	9.00	10	0.53
Old age psychiatry, *n* (%)	9 (14.3)	10 (18.5)	8 (14.5)			
Child and adolescent psychiatry, *n* (%)	5 (7.9)	7 (13.0)	5 (9.1)			
Intellectual disability psychiatry, *n* (%)	4 (6.3)	4 (7.4)	1 (1.8)			
Forensic psychiatry, *n* (%)	14 (22.2)	11 (20.4)	8 (14.5)			
Medical psychotherapy, *n* (%)	2 (3.2)	2 (3.7)	0 (0.0)			

PMQ, primary medical qualification; IMG, international medical graduate; EEA, European Economic Area.

There has been no change in the proportion of women in senior psychiatric leadership roles: in fact, in absolute terms there are four fewer female doctors in such roles compared with 2016. There has been a substantial increase in the proportion of medical directors who are international medical graduates, with all of them originating in either South Asia (12 India, 4 Pakistan) or Africa (4 Nigeria, 2 South Africa, 1 Zimbabwe, 1 Ghana). There were no significant differences in characteristics by specialist registration or by years from PMQ to board appointment.

The majority (32 out of 55) of board-level psychiatrists have taken up their appointment since July 2020. The median year for board appointment for the whole sample is 2021. Comparing those appointed since 2021 with those appointed earlier, psychiatrists appointed recently have taken longer to reach board appointment from PMQ (pre-2021: mean 22.6 years; post-2021: mean 26.0 years; *t* = 2.01, d.f. = 53, *P* = 0.03). There were no statistically significant differences in characteristics by sex, PMQ region or specialist registration. However, in absolute numbers more women have been appointed in the past 3 years (10 since 2021; 5 prior to 2021). Similarly, there has been a growth in the number of general adult psychiatrists appointed recently (20 since 2021, 13 prior to 2021).

**Fig. 1:**
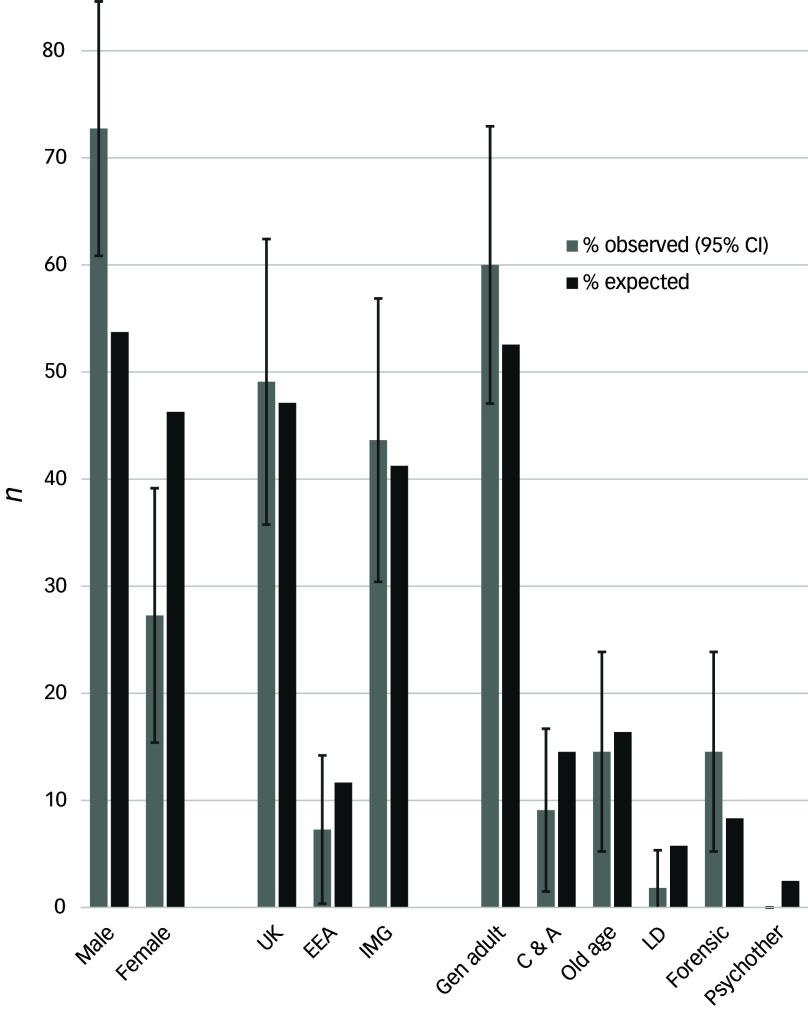
Expected and observed characteristics of board-level psychiatrists in England’s mental health trusts in 2024 by sex, country of primary medical qualification and psychiatric specialty. EEA, European Economic Area; IMG, international medical graduate; Gen adult, general adult psychiatry; C & A, child and adolescent psychiatry; LD, intellectual (learning) disability psychiatry; Psychother, medical psychotherapy.

The differences between observed and expected proportions are shown in Fig. 1. Men are overrepresented (*z* = 2.80, *P* = 0.005). There is no difference between observed and expected proportions with respect to PMQ region. Intellectual disability psychiatrists are underrepresented (*z* = 3.40, *P* = 0.001). There are fewer child and adolescent psychiatrists and medical psychotherapists than expected, and more forensic psychiatrists than expected. However, given the small numbers, these differences do not reach statistical significance.

## Discussion

Our study highlights several significant findings regarding the characteristics of medical directors in mental health trusts in England between 2016 and 2024. One of the most notable observations is the substantial turnover in medical director positions. This turnover has been accompanied by an increase in the representation of international medical graduates (IMGs) among medical directors. However, despite this increase in diversity, the representation of women in these roles has not seen a similar increase. Women remain underrepresented, although there are potential signs of change, with recent appointments showing a slight upward trend. The number of intellectual disability psychiatrists at board level has reduced substantially, although the numbers are small anyway.

The difficulty that women doctors have in career progression in the NHS has long been noted, with this first being attributed in part to the likelihood of them not working full-time throughout their career.^14^ There is evidence that during the COVID-19 pandemic cultural changes were made that influenced new styles of working more suited to the needs of female employees, with increased opportunities for flexible working patterns, including working from home.^15^ Research into methods of better promoting female career progression to leadership roles highlights the importance of flexible work policies and change in the wider organisational structure to decrease the barriers in place.^4^ But most recently, concern has been expressed that negative perceptions of women as leaders in healthcare and beyond are increasing and that multiple factors can influence retention of women in medical leadership roles.^16,17^ This can include ingrained gender stereotyping in professional environments and difficulty in balancing personal life commitments with work, but individual circumstances vary greatly and are usually subject to multiple factors.^18^

The change in IMG representation appears to be influenced by both the retention of older IMGs and the onboarding of new IMGs, who now constitute 44% of the group. Interestingly, newer IMGs are taking longer to secure board appointments, suggesting possible barriers or differences in the integration process that warrant further exploration.

These findings are particularly significant in the context of the current NHS landscape, where diversity and inclusivity are critical for effective leadership and service delivery. The increasing representation of IMGs is a positive step towards a more diverse leadership, yet the persistent underrepresentation of women psychiatrists indicates that more concerted efforts are needed to achieve equitable representation.

### Strengths and limitations

One of the strengths of this study is the completeness of the data-set, which provides a comprehensive overview of the characteristics of board-level psychiatrists over the specified period. However, a limitation is the inability to examine protected characteristics using the available data. This constraint prevents a more granular analysis of diversity and inclusivity within senior medical leadership roles.

### Implications

Future research should focus on monitoring changes in the characteristics of senior psychiatric leaders in the coming years to assess whether the observed trends continue. But more than simply highlighting the trends at board level, we need to examine representation in the tiers of medical management below board level, as that is where medical directors are recruited from. The importance of medical leadership development for all psychiatrists has been emphasised,^19^ and we should monitor the characteristics of those accessing formal leadership training at a local, regional and national level. Opportunities need to be available to early career psychiatrists in both training and in non-training grades and across the psychiatric specialties. We need to ensure that the specialty career choice psychiatrists make does not limit their leadership potential.

## Data Availability

Data are available from the corresponding author.

## About the authors

**Peter L. Cornwall**, MD, FRCPsych, FFMLM, is a consultant psychiatrist with Redcar & Cleveland Mental Health Services, Tees, Esk & Wear Valleys NHS Foundation Trust, Redcar, UK. **Penny Walton** is a medical student at Newcastle University Medical School, Newcastle upon Tyne, UK.
